# Treatment of Helminth Co-Infection in Individuals with HIV-1: A Systematic Review of the Literature

**DOI:** 10.1371/journal.pntd.0000102

**Published:** 2007-12-19

**Authors:** Judd L. Walson, Grace John-Stewart

**Affiliations:** Department of Medicine, University of Washington, Seattle, Washington, United States of America; London School of Hygiene & Tropical Medicine, United Kingdom

## Abstract

**Background and Objectives:**

The HIV-1 pandemic has disproportionately affected individuals in resource-constrained settings. It is important to determine if other prevalent infections affect the progression of HIV-1 in co-infected individuals in these settings. Some observational studies suggest that helminth infection may adversely affect HIV-1 progression. We sought to evaluate existing evidence on whether treatment of helminth infection impacts HIV-1 progression.

**Review Methods:**

This review was conducted using the HIV/AIDS Cochrane Review Group (CRG) search strategy and guidelines. Published and unpublished studies were obtained from The Cochrane Library (Issue 3, 2006), MEDLINE (November 2006), EMBASE (November 2006), CENTRAL (July 2006), and AIDSEARCH (August 2006). Databases listing conference abstracts and scanned reference lists were searched, and authors of included studies were contacted. Data regarding changes in CD4 count, HIV-1 RNA levels, clinical staging and/or mortality were extracted and compared between helminth-treated and helminth-untreated or helminth-uninfected individuals.

**Results:**

Of 6,384 abstracts identified, 15 met criteria for potential inclusion, of which 5 were eligible for inclusion. In the single randomized controlled trial (RCT) identified, HIV-1 and schistosomiasis co-infected individuals receiving treatment for schistosomiasis had a significantly lower change in plasma HIV-1 RNA over three months (−0.001 log_10_ copies/mL) compared to those receiving no treatment (+0.21 log_10_ copies/mL), (p = 0.03). Four observational studies met inclusion criteria, and all of these suggested a possible beneficial effect of helminth eradication on plasma HIV-1 RNA levels when compared to plasma HIV-1 RNA changes prior to helminth treatment or to helminth-uninfected or persistently helminth-infected individuals. The follow-up duration in these studies ranged from three to six months. The reported magnitude of effect on HIV-1 RNA was variable, ranging from 0.07–1.05 log_10_ copies/mL. None of the included studies showed a significant benefit of helminth treatment on CD4 decline, clinical staging, or mortality.

**Conclusion:**

There are insufficient data available to determine the potential benefit of helminth eradication in HIV-1 and helminth co-infected adults. Data from a single RCT and multiple observational studies suggest possible benefit in reducing plasma viral load. The impact of de-worming on markers of HIV-1 progression should be addressed in larger randomized studies evaluating species-specific effects and with a sufficient duration of follow-up to document potential differences on clinical outcomes and CD4 decline.

## Author Summary

Many people living in areas of the world most affected by the HIV/AIDS pandemic are also exposed to other common infections. Parasitic infections with helminths (intestinal worms) are common in Africa and affect over half of the population in some areas. There are plausible biological reasons why treating helminth infections in people with HIV may slow down the progression of HIV to AIDS. Thus, treating people with HIV for helminths in areas with a high prevalence of both HIV and helminth infections may be a feasible strategy to help people with HIV delay progression of their disease or initiation of antiretroviral therapy. After a comprehensive review of the available literature, we conclude that there is not enough evidence to determine whether treating helminth infections in people with HIV is beneficial.

## Introduction

Many individuals living in areas of the world hardest hit by the HIV-1 epidemic are also infected with other common pathogens. These infections may have detrimental effects on the host's ability to control the HIV-1 virus [1]. Some studies have suggested that these infections may result in a more rapid progression of HIV-1 disease [2]. Co-infection with other pathogens may lead to a more rapid destruction of the host immune system and potentially to earlier progression of HIV-1. Chronic helminth infection may suppress immune responses directed against HIV-1 and concurrent immune activation may directly lead to more rapid loss of CD4 cells in HIV-1 infected individuals [3].

Of the over 25 million people infected with HIV-1 in Africa, it is estimated that as many as half of these individuals may be co-infected with helminths [4],[5]. Helminth infection leads to significant stimulation of the host immune response as these infections are characterized by the production of eggs, excretory products, and secretions. Helminth-infected individuals display increased levels of eosinophilia, increased IgE levels, and a Th2 immune bias [6],[7]. Immunoregulation in response to helminth infection may suppress HIV-1-specific CD4+ and CD8+ proliferation and cytokine production which may compromise control of HIV-1 replication [8]. Chronic helminth infection has also been shown to be associated with antigen-specific anergy and hyporesponsiveness which may also down-regulate control of HIV-1 replication [9]. Immune activation may also result in increased cellular susceptibility to HIV-1 infection [10].

Several studies have suggested that helminth co-infection in HIV-1 infected individuals may result in increased plasma levels of HIV-1 RNA and possibly in more rapid disease progression [2],[11]. Based on these findings, the hypothesis that helminth infection may play an important role in the pathogenesis of HIV-1 in Africa was suggested by Bentwich et al [12].

In a study of HIV-1 infected and uninfected individuals in Ethiopia, helminth co-infection was associated with increased T-cell activation and anti-helminthic treatment appeared to reduce T-cell activation. In addition, treatment of helminth infection resulted in a significant increase in absolute CD4 counts (192 versus 279 cells/mm^3^, p = 0.002) [7]. Another study conducted in Ethiopia noted an association between stool helminth burden and plasma HIV-1 RNA levels among individuals with helminth co-infection (p<0.001). Successful treatment of helminth co-infection (clearance of helminth eggs in stool) led to a significant decrease in HIV-1 plasma viral load (−0.36 log_10_) in these patients [2]. However, several subsequent observational studies have shown conflicting results regarding the impact of anti-helminth therapy on CD4 count, HIV-1 viral load and clinical disease progression [13],[14],[15].

As HIV-1 treatment programs are expanded in areas in which both HIV-1 and helminth infections are prevalent, it is important to determine whether treating helminth infection can slow HIV-1 disease progression. Mathematical modeling of potential HIV-1 vaccine efficacy suggests that even a modest 0.5 log reduction in set-point HIV-1 RNA levels could slow the onset of AIDS by 3.5 years and could delay the need for antiretroviral medications by almost a full year [16]. If anti-helminthic therapy enables HIV-1 infected individuals to delay initiation of antiretroviral therapy or reduce morbidity and mortality, the public health significance of de-worming HIV-1 infected individuals may be substantial.

In this systematic review we evaluated the evidence to date that treating helminth infection in HIV-1 and helminth co-infected individuals may impact HIV-1 progression by decreasing HIV-1 virus (HIV-1 RNA), attenuating CD4 decline, or delaying the onset of symptoms of AIDS.

## Methods

We developed a protocol for this review for inclusion in the Cochrane Database of Systematic Reviews [17]. We included randomized or quasi-randomized controlled trials assessing the association between helminth co-infection and HIV-1 disease progression. We pre-specified that should data from clinical trials be insufficient, data from observational studies (e.g. cohort, case-control and cross-sectional studies) would be considered for inclusion in this review according to the HIV/AIDS CRG policy. Both interventional clinical trials as well as observational case control and cohort studies of HIV-1 and helminth co-infected individuals were included. Studies evaluating the effect of anti-helminthic therapy on HIV-1 progression were identified. Anti-helminthic therapy was defined as any intervention approved for use in the eradication of helminth infection in humans. This included the benzimidazoles, ivermectin, praziquantel, diethylcarbamazine, bithionol, oxamniquine, pyrantel and nitazoxanide. Control groups included placebo, no treatment, or helminth uninfected individuals. Studies evaluating changes in CD4 counts and/or HIV-1 viral load before and after anti-helminthic therapy were also included. We included studies performed in general or specific populations, in both hospitals and/or clinics, in any country and published in any language. Ecological studies were excluded. The full text of the search strategy employed has been published previously [17]. The search was conducted by two reviewers (JW and GJS). There were no disagreements in applying the inclusion criteria for selected studies.

The primary outcome measures selected were changes in plasma HIV-1 RNA levels and changes in absolute CD4 counts. Secondary outcome measures included markers of clinical disease progression, adverse events, and mortality.

The methodological quality of the included clinical trial was evaluated independently by both authors (JW and GJS) according to a validity checklist for clinical trials[18]. The methodological quality of the included observational studies was assessed using the Newcastle-Ottawa Quality Assessment Scales for observational studies (Table 1) [19]. Adequacy of follow up was assessed as adequate (trials with loss to follow up ≤20%), inadequate (loss to follow up above 20%) or unclear (not reported). All outcomes included in this review were continuous and were assessed with a standardized mean difference (SMD) and 95% confidence interval. A narrative synthesis was performed. We did not statistically pool the outcomes and examine the differences between fixed and random effects models given the degree of heterogeneity between the trials.

**Table 1:** Assessment of quality of cohort studies

Study	External Validity	Performance Bias	Detection Bias	Attrition Bias	Selection Bias
Description of assessment criteria	How was sampling conducted (census, random, etc.) and were at least 80% of those eligible to participate in all groups recruited?	Was the same method of ascertainment used in cases and controls?	How were HIV-1 and helminth infection status ascertained and confirmed and were assessors of outcome measures blinded to intervention groups?	Were all groups followed for the same time frame and were at least 80% of participants in all groups included in the final analysis?	How were cases selected and were controls selected from the same population as the cases?
Brown 2004	**	**			
Elliott 2003	**	**	**	**	
Wolday 2002	**	**	**		
Modjarrad 2005	**	**		**	

**: study design adequate to minimize role of bias

## Results

**Figure 1:**
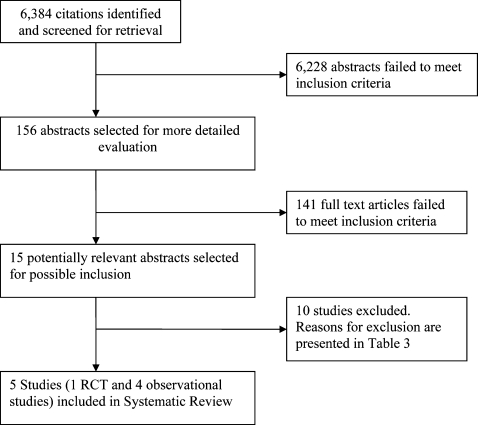
Flow diagram of study selection process in QUOROM format.

After an expanded search strategy was employed, we identified 6,384 citations. From this list, we identified 15 potentially relevant studies, of which 5 were determined to be eligible for this review, including one randomized controlled trial and four observational studies (Figure 1). The characteristics of the included studies are presented in Table 2. All of the included studies were conducted in Africa and included HIV-1 infected individuals who were treated for a variety of different helminth infections. Four of the five studies noted that none of the participants were on antiretroviral medication while in one study [13], data on antiretroviral therapy was not provided. In the observational studies, changes in HIV-1 RNA or CD4 were compared between helminth-treated individuals and helminth uninfected controls, or controls in the period prior to helminth treatment. Three studies also compared changes in HIV-1 RNA or CD4 between treated individuals who cleared their helminth infection at follow-up and individuals who remained infected at follow-up. Unpublished data was requested from the authors of the included observational studies and included in this analysis from three of included observational studies [11],[13],[14].

**Table 2:** Characteristics of included studies

Study	Methods	Participants	Interventions	Outcomes	Helminth Species Included (% of total infections)	Notes
Brown 2004	Cohort of HIV-1 infected subjects in Entebbe, Uganda. All subjects had duplicate Kato-Katz smears, modified formol-ether concentrations and charcoal cultures performed on stool samples. ELISA for circulating anodic antigen (CAA) of *Schistosoma mansoni* was performed on serum samples. Subjects were also screened for microfilaria and *Wuchereria bancrofti.* All subjects were followed one month after enrollment and at their routine 6 month visit.	Adults attending the AIDS Support Organization Clinic of the UK Medical Research Council Entebbe Cohort in Entebbe, Uganda. 663 subjects were recruited and this analysis included data on the 177 subjects who had available CD4 counts from 6 months prior to enrollment, enrollment and the 6 month follow up visit.	All subjects received albendazole 400 mg directly observed at enrollment. Those participants found to be infected with schistosomes were treated with a single dose of praziquantel 40 mg/kg.	The effect of helminth infection and treatment on changes in CD4 count and HIV-1 RNA in HIV-1 infected participants.	*Schistosomiais mansoni* (31.4%) Hookworm (21.9%) *Strongyloides stercoralis* (12.4%) *Mansonella perstans* 7.5%) *Trichuris trichiura* (3.7%) *Ascaris lumbricoides* (1.1%) *Trichostrongylus sp.* (0.5%) Mixed infection (34%)	This study used data from the same cohort as the Elliott 2003 study. The >20% drop out rate seen in this study reflects 58 individuals who did not provide sufficient stool for charcoal culture (to determine Strongyloides infection). The excluded subjects did not significantly differ at baseline from the included subjects. Additional CD4 and HIV-1 RNA data were provided by the authors.
Elliott 2003	Cohort study of HIV-1 seropositive subjects in Uganda. Subjects attending routine appointments were recruited. All subjects had stool samples examined using Kato-Katz method for helminths and underwent serum ELISA for circulating anodic antigen (CAA) for detection of *Schistosoma mansoni*. All subjects were followed at 5 weeks and 4 months after enrollment.	Adults attending the AIDS Support Organization Clinic of the UK Medical Research Council Entebbe Cohort in Entebbe, Uganda. 120 HIV-1 seropositive individuals were enrolled and 39 HIV-1 and helminth co-infected individuals were included in this analysis.	Nematode infected individuals were treated with mebendazole 100mg twice a day for three days and schistosomiasis was treated with two doses of 20mg/kg of praziquantel.	Change in CD4 count in the 6 months prior to treatment compared to change in CD4 count between enrollment and the 4 month follow up visit	*Schistosomiais mansoni* (71.8%) Hookworm (38.5%) *Trichuris trichiura* (15.4%) *Ascaris lumbricoides* (5.1%) *Taenia sp.* (2.6 %) Mixed infection (Data not provided)	This study used data from the same cohort as the Brown 2004 study. The retrospective comparison of CD4 data was not included in the paper but the unpublished data were provided by the authors.
Kallestrup 2005	Randomized, unblinded, controlled trial of immediate versus delayed (at 3 months) therapy of schistosomiasis. Patients with and without HIV-1 infection who were found to be infected with schistosomes were randomized to receive praziquantel at enrollment or after a delay of three months. Data from the HIV-1 seropositive cohort are included in this analysis.	Adult participants were recruited through community meetings in rural Zimbabwe. 287 individuals were enrolled of whom 130 with HIV-1 and schistosome co-infection were included in this analysis. 64 participants received early treatment and 66 received delayed treatment.	Participants received praziquantel 40mg/kg either at enrollment of after a delay of 3 months.	Changes in plasma HIV-1 RNA levels and CD4 count between individuals randomized to early versus delayed treatment (3 months later).	Schistosomiasis (100%)	Unblinded RCT conducted in rural Zimbabwe. Randomization method not specified and no allocation concealment
Modjarrad 2005	Prospective cohort trial examining the impact of antihelminthic treatment on HIV-1 RNA concentrations in Zambia. The primary objective of the study was to assess differences in HIV-1 RNA levels between HIV-1 and helminth co-infected individuals who were treated with anti-helminthics and HIV-1 infected, helminth uninfected individuals who did not receive antihelminthics.	The investigators screened 428 adults for HIV-1 serostatus and assessed helminth infection using stool microscopy. 54 HIV-1/helminth co-infected individuals were recruited into the study and matched by sex and age (±4 yrs) with HIV-1 infected, helminth uninfected participants.	Co-infected individuals were treated at week 1 and week 4 following enrolment with albendazole (400 mg on the first day and 200 mg for 2 subsequent days) and praziquantel 40 mg/kg divided into 2 doses given 4-6 hours apart). HIV-1 infected, helminth uninfected individuals were not treated.	Participants were followed at 4 weeks, 10 weeks and 16 weeks following the initiation of treatment. HIV-1 RNA and CD4 counts were measured at baseline, week 1, week 10 and week 16.	*Ascaris lumbricoides* (48.1%) Hookworm (33.3%) *Schistosomiais mansoni* (9.3%) *Strongyloides stercoralis* (7.4%) *Hymenolepsis nana* (1.9%) Mixed infection (Data not provided)	Not all participants received anti-helminthic therapy
Wolday 2002	Prospective cohort trial of HIV-1 infected individuals in Ethiopia. Participants were treated regardless of stool study findings at enrollment and at 3 and 6 months of follow up. Two stool samples collected at least 4 hours apart were screened using Kato-Katz technique and by formalin-ether concentration. Six smears were prepared from each stool sample. Participants were followed up at 3 and 6 months visits following enrollment.	56 consecutive ambulatory asymptomatic HIV infected subjects in Ethiopia were recruited. All subjects were asymptomatic and had never taken antiretrovirals. At baseline, 31 individuals had helminth infection with one or more species.	All participants were treated at enrollment and at the 3 and 6 month follow up visit with 200 mg per day of albendazole for 3 days. Those found on stool examination to have schistosomiasis were also were treated with 40mg/kg of praziquantel.	Participants were followed at 3 and 6 months following enrollment. HIV-1 RNA and CD4 counts were measured at baseline and at the 3 month and 6 month follow up visits.	*Trichuris trichiura* (45.2%) *Ascaris lumbricoides* (41.9%) *Schistosomiais mansoni* (12.9%) *Strongyloides stercoralis* (3.2%) Mixed infection (Data not provided)	HIV RNA measurements were available at enrollment and the 6 month follow up visit for only 28 individuals (50%). Comparisons were made between the groups infected at baseline and helminth uninfected individuals as well as between those infected at baseline who were helminth negative at follow up and those who remained helminth infected at follow up.

The ten excluded studies along with the rationale for exclusion are presented in Table 3 [1],[7],[18],[20],[21],[22],[23],[24],[25],[26]. Reasons for exclusion included inadequate reporting or collection of outcome data [1],[7],[18],[20],[21],[22],[25],[26], lack of a control comparison group [1],[22],[26], failure to confirm helminth infection status[18], and reporting of data already presented in another included study [23].

**Table 3:** Characteristics of excluded studies

Study	Reason for exclusion	Findings
Brown 2005	No comparator group. All patients treated with albendazole prior to enrollment and those with schistosomiasis treated with praziquantel at enrollment. No data on changes prior to treatment (other than the 1 month prior to treatment with albendazole) presented. No uninfected or infected and untreated comparator group available.	163 Ugandan adults with HIV-1 and *Schistosoma mansoni* co-infection were treated with praziquantel and followed for 5 months. There was no significant change in CD4 counts at 1 month but a significant decline was noted at the 5 month follow up visit (p<0.01). A statistically significant increase in HIV-1 RNA levels was noted at one month (4.76 to 4.89 log_10_ HIV-1 RNA, p = 0.001) but not at 5 months (values not reported).
Gallagher 2005	No data on maternal viral load, CD4 counts or clinical progression were available. The authors were contacted and stated that these data were not collected.	83 pregnant HIV-1 infected women were screened for parasite co-infection and compared to 166 HIV-1 uninfected pregnant women. There was no significant difference noted in baseline intestinal helminth or schistosomiasis prevalence between the groups. Eleven of 23 women with one or more helminth infection transmitted HIV-1 to their infant (48%) compared to two of twenty women (10%) who did not have helminth co-infection (p<0.01). HIV-1 infected women with helminth co-infection were 7 fold more likely to transmit HIV-1 to their infant after controlling for the effect of concurrent malaria infection. Data on maternal HIV-1 RNA or CD4 counts were not presented.
Ganley-Leal 2006	No data on CD4, HIV-1 RNA or clinical progression after treatment of schistosomiasis were presented.	This study did not present comparative data evaluating CD4, HIV-1 RNA or clinical progression in the context of schistosomiasis infection.
Hosseinipour 2007	Individuals with both protozoal and helminth infection included. Follow up was only conducted on treated individuals, no data available for comparison in change in CD4 or HIV-1 RNA between treated and untreated or helminth infected and helminth uninfected comparator groups.	Among 266 HIV-1 infected individuals in Malawi, 35 had at least one helminth infection (29 with geohelminth infection and 6 with schistosomiasis). Baseline HIV-1 RNA levels were not significantly different between the group with helminth co-infection and helminth uninfected individuals. Baseline median CD4 counts were significantly lower among patients without a helminth infection (235 cells/µL) compared to those with a helminth infection at baseline (320 cells/µL), (p<0.001). Four weeks following treatment of helminth infection, there was no significant change in HIV-1 RNA levels in these individuals (p = 0.86).
Kallestrup 2006	This manuscript presents further analyses using data already presented in Kallestrup 2005.	Relevant data was presented in the included RCT (Kallestrup 2005)
Kassu 2003	Authors report changes in CD4 counts in a group treated for helminths but do not specify treatments. Control group is a group of participants who were uninfected initially but developed infection in the 6 months prior to repeat testing. No data on standard deviation of CD4 results available and not enough information provided to calculate.	Peripheral blood mononuclear cells (PBMC's) from 64 Ethiopian adults (41 HIV-1 uninfected and 23 HIV-1 infected) were tested at baseline and at 6 month follow up visits. Among the 23 HIV-1 infected individuals, 16 were co-infected at baseline with at least one helminth. There was no significant difference in baseline CD4 counts among HIV-1 infected individuals when compared by helminth infection status. Treatment of helminth co-infection did not result in significant changes in absolute CD4 counts among the HIV-1/helminth co-infected cohort. Incidental helminth infection occurring during the study (in both HIV-1 infected and uninfected participants) appeared to increase memory CD4 counts (p = 0.03) and memory CD8 counts (p = 0.02) and this appeared to be due mainly to infection with *Ascaris lumbricoides* infections (data not shown).
Kelly 1996	This study did not perform any testing to confirm the presence of helminth infection. In addition, outcome data did not include HIV-1 RNA, CD4 counts or markers of clinical progression.	This study did not present comparative data evaluating CD4, HIV-1 RNA or clinical progression in the context of documented helminth infection.
Lawn 2000	No comparison group. Participants enrolled and treated. Changes in HIV-1 RNA and CD4 then recorded over time. No information presented on differences in outcome measures between successfully treated individuals and those who were not successfully cured of schistosomiasis or who were re-infected.	30 individuals in Kenya with documented HIV-1 and Schistosomiasis co-infection were enrolled and treated with praziquantel. Comparisons of pretreatment and follow up samples (mean follow-up of 5.6 months, range 1–15 months) revealed an increase in mean plasma HIV-1 RNA (3.60 ±0.90 to 3.93 ± 0.95 log_10_ HIV-1 RNA, p<0.001).
McElroy 2005	This study compared CD4 and Viral load in HIV-1 infected individuals with and without schistosomiasis infection. Participants did not receive any intervention as part of this analysis and so were not assessed before and after treatment of helminths.	35 HIV-1 infected Ugandan adults were included in this analysis. Twelve of these individuals had confirmed *Schistosoma mansoni* infection. There were no significant differences in baseline CD4 counts or HIV-1 RNA levels between the *S. mansoni* infected group (CD4 count 212 cells/µL, HIV-1 RNA 97.2×10^3^ copies/mL) and the *S. mansoni* uninfected group (CD4 count 230 cells/µL, HIV-1 RNA 126.1×10^3^ copies/mL), (p = 0.98 for CD4 comparison, p = 0.8 for HIV-1 RNA comparison).
Mwanakasale 2003	This manuscript did not report changes in CD4 counts, HIV-1 RNA levels or clinical progression markers.	This study did not present comparative data evaluating CD4, HIV-1 RNA or clinical progression in the context of schistosomiasis infection.

The studies were stratified according to study type, i.e. RCT vs. observational. We had pre-specified in our protocol that meta-analysis would be performed if data permitted. Given that only one RCT was identified, the highly heterogeneous comparator groups and results, as well as the high likelihood of bias in the observational studies, it was felt that any overall summary statistic would be misleading and a meta-analysis was not performed. Instead, we evaluated the direction and consistency of effect, assessing the likelihood of bias for each included study and investigating factors that may explain the differences observed between the studies. Studies were evaluated using the standardized mean difference (SMD = Difference in mean outcome between groups/Standard deviation of outcome among participants) [27].

**Figure 2:**
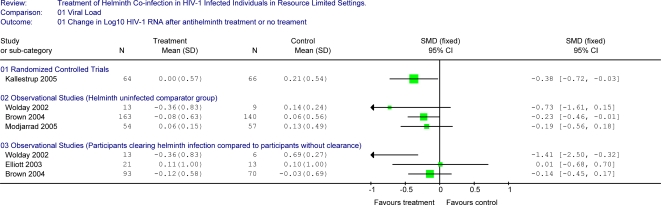
Forest plot showing changes in Log_10_ HIV-1 RNA between treatment group and selected comparator groups.

**Figure 3:**
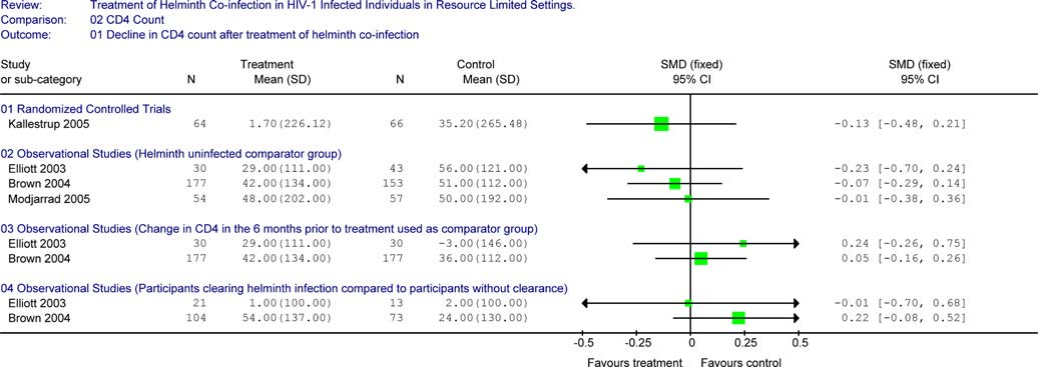
Forest plot showing changes in CD4 counts between treatment group and selected comparator groups.

The results of the single randomized control trial demonstrated a statistically significant benefit on plasma HIV-1 RNA levels with treatment of schistosomiasis co-infection in HIV-1 infected adults compared to no treatment over 3-months of follow-up (Figure 2) [11]. Individuals in the treatment group had minimal change in plasma viral load over three months of follow up (−0.001 log_10_ copies/mL) compared to an increase in those who did not receive treatment during this period (0.21 log_10_ copies/mL), (p = 0.03). This trial noted a statistically significant benefit on CD4 count with treatment when both HIV-1 infected and HIV-1 uninfected individuals were included, with a non-significant trend for a difference between the two study arms when limited to HIV-1 infected individuals (treatment resulted in a 1.7 cells/µL mean decline compared to a 35.2 cells/µL mean decline in the untreated group (p = 0.17) (Unpublished data provided by author)) (Figure 3). This study also evaluated differences in CDC clinical staging between the treated and untreated group. At the 3-month visit, there were no differences with regard to the number of individuals in CDC stage A, B or C (43∶20∶1 for the treatment arm compared to 44∶20∶2 in the untreated arm) although the study was underpowered for this assessment.

We stratified the four additional observational studies by outcome variable (HIV-1 RNA or CD4 count) and by comparator group (helminth-uninfected controls, same individuals during interval prior to helminth treatment, or individuals who failed to clear helminths at follow-up).

### Study Results Comparing Changes in HIV-1 RNA Levels

#### Helminth-treated versus helminth-uninfected controls

Three observational studies presented data comparing treated HIV-1 helminth co-infected individuals to HIV-1 infected helminth-uninfected controls [2],[13],[15] (Figure 2). The results of all of these studies were in the direction of a benefit in reducing plasma HIV-1 RNA viral load with treatment of helminth co-infection. In the study by Brown et al, participants were screened for helminths and treated with albendazole regardless of helminth infection status. In the other 2 studies, helminth-uninfected participants did not receive anti-helminthics. Treatment of helminth infected participants in the Modjarrad study included both albendazole and praziquantel. The helminth-treated cohort size in these studies ranged from 13 to 163 participants and the helminth-uninfected cohort size ranged from 9 to 140 participants. Follow-up was between 3 and 6 months. Helminth-treated participants had HIV-1 RNA changes ranging from −0.36 log_10_ to +0.06 log_10_ copies/ml following treatment; the standard mean difference in HIV-1 RNA between helminth-treated and the helminth-uninfected individuals followed over the same period ranged from −0.19 to −0.73, all in the direction of decreased HIV-1 RNA or lower plasma HIV-1 RNA increase in helminth-treated individuals compared to helminth-uninfected comparators.

#### Participants clearing infection at follow up compared to participants with continued infection at follow up

Three observational studies compared individuals who cleared their initial helminth infection following treatment to those who were helminth-infected at follow-up (Figure 2) [2],[13],[14]. Two studies showed a trend towards a beneficial effect on plasma viral load with the successful treatment of helminth infection while one study did not. These cohorts included between 13 and 93 individuals who cleared their helminth infection and between 6 and 70 comparators who failed to clear helminth infection. Change in plasma HIV-1 RNA ranged from −0.36 to +0.11 log_10_ copies/ml during 3 to 6 months of follow-up in those who cleared their helminth infection versus changes of −0.03 to +0.69 log_10_ copies/ml in those who did not (standard mean difference of −0.14 to −1.41).

### Study Results Comparing Changes in CD4 Counts

#### Helminth-uninfected controls

Changes in CD4 counts were compared between individuals treated for helminth infection and individuals who were not infected with helminths at baseline in 3 studies (Figure 3) [13],[14],[15]. While there appeared to be a beneficial effect of treatment on CD4 count in all of these studies, none were statistically significant. Over 3–6 months of follow-up, helminth-treated individuals experienced mean decreases in CD4 counts of 29–48 cells/µL (cohorts of between 30 and 177 individuals) while decreases among individuals without helminth infection ranged from 50–56 cells/µL over the same period (cohorts of between 43 and 153 individuals). The standardized mean difference ranged from −0.01 to −0.23 for these comparisons.

#### Changes in CD4 counts within individuals prior to and after helminth eradication therapy

Two observational studies compared changes in CD4 counts in a 6 month period prior to treatment of helminths to changes observed during follow-up post-treatment in the same individuals [13],[14]. Neither of these studies showed significant differences in CD4 count changes before and after treatment of helminth co-infection. In the 6 month period preceding helminth treatment, one study noted a CD4 decline of 36 cells/µL in 177 individuals and a decline of 42 cells/µL in the 6 months following treatment while the other study documented a rise in mean CD4 count of 3 cells/µL among 30 individuals prior to treatment versus a decline of 29 cells/µL following treatment (unpublished data provided by authors).

#### Participants clearing infection at follow up compared to participants with continued infection at follow up

Changes in CD4 counts between helminth co-infected individuals who cleared their infection following treatment and those who were infected with helminths at the follow-up visit were compared in two studies [13],[14]. In contrast to the expected direction of effect, one study noted that among 104 individuals who cleared their helminth infection, there was a mean decline of 54 cells/µL compared to a decline of 24 cells/µL among 73 individuals with persistent helminth infection (p = 0.14). In another study, 21 individuals who cleared their helminth infection had a median increase in CD4 count of 1 cells/µL compared to an increase of 2 cells/µL in 13 individuals with persistent infection (unpublished data provided by authors).

#### Mortality

Mortality data were presented in three of the included studies. In one study, there were 13 deaths over 6 months of follow-up among 144 helminth-treated individuals (mortality rate of 90.4 cases/1000 person-years, 95% CI 52.5–155.6) compared to 13 deaths among 122 helminth-uninfected individuals (mortality rate 106.4 cases/1000 person years, 95% CI 61.8–183.3), (difference in mortality, p = 0.68) [13]. In a smaller study, there were 2 deaths among the 31 treated helminth-infected individuals and 2 deaths among the 25 helminth-uninfected individuals [2]. Finally, in a third study there was 1 death in each of the groups (helminth-treated and helminth-uninfected) [15].

#### Helminth species-specific effects

The RCT was the only study designed to evaluate the effect of treating a specific helminth (schistosomiasis). There were differences in species-specific prevalence rates reported in each of the included studies (Table 2). The studies by Elliott et al, Brown et al and Modjarrad et al reported differences between helminth species although all were underpowered to detect potentially meaningful effects by individual species. Elliott et al also reported a significant decline in log_10_ HIV-1 RNA levels among individuals with schistosomiasis (n = 24) between 5 weeks following therapy and the 4 month follow-up visit (p = 0.01). In contrast to this, Brown et al reported significantly greater decreases in CD4 counts in individuals who successfully cleared their schistosomiasis infection at 6 months (n = 105, mean decrease of 59.8 cells/µL) compared to those who remained persistently infected with schistosomes (n = 54, mean decrease of 15.5 cells/µL) (p = 0.05), Brown et al also found that among *Mansonella perstans* infected subjects, persistent infection at follow up (n = 22) was associated with a decrease from 4.86 to 4.67 log_10_ HIV-1 RNA (p = 0.009).

## Discussion

It has been suggested that de-worming is unlikely to have an effect on HIV-1 progression in co-infected individuals [22]. However, to date there has been only one randomized controlled trial evaluating the potential benefit of treating helminth co-infection in HIV-1 infected individuals which was limited to evaluation of schistosomiasis co-infection. The results of our systematic review including the single randomized clinical trial and four observational studies demonstrate that there are insufficient data regarding the potential impact of de-worming on HIV-1 progression in co-infected individuals.

The only RCT conducted to date demonstrated a statistically and clinically significant difference in change in HIV-1 RNA levels (0.21 log_10_ HIV-1 RNA) in treated versus untreated individuals with schistosomiasis [11]. This study failed to show significant differences in changes in CD4 counts or clinical staging between groups. The study was limited by a lack of allocation concealment, a short follow-up interval (3 months), and differences at baseline in gender distribution, CD4 counts and plasma HIV-1 viral loads between randomization groups. In the 4 observational studies eligible for inclusion in this review, there was also a trend towards a beneficial effect of helminth treatment on HIV-1 RNA levels in co-infected individuals. Again, de-worming did not appear to exert a benefit on CD4 count decline or mortality in these studies, and some studies found greater CD4 decline following successful helminth clearance when compared to persistently infected individuals. Together, the cumulative available evidence suggests a possible benefit of de-worming on plasma HIV-1 RNA levels but not CD4 counts or clinical status over a short period of follow-up.

In the observational studies reviewed, groups of helminth-treated individuals were compared to 3 different types of “control” groups, all of which were likely to differ at baseline from the group with helminth infection in which the intervention was administered. Helminth-infected individuals may have pre-existing differences in behavioral, social, nutritional and biological factors that led to helminth infection and may also result in faster HIV-1 progression. A comparator group of helminth co-infected individuals who did not clear their helminth infection is similarly subject to bias, as helminth clearance may be directly related to the capacity of the host immune system to handle such infections, which may also correlate with control of HIV-1. Comparing individual data from a period of time prior to de-worming to data following therapy is also subject to limitations. Individuals may acquire helminth infection during the period between the initial measurement of CD4 count or HIV-1 RNA and the de-worming visit and this could result in misclassification of the exposure. In addition, rates of change in CD4 count and viral load in HIV-1 infected individuals differ over time, making it difficult to compare changes in these parameters at two different periods within an individual. These limitations compromise the strength of the evidence regarding treatment effect and highlight the need for randomized placebo-controlled trials to determine effects of anti-helminthics on HIV-1 progression.

There are important possible interactions between helminths and HIV-1 that may be dependent on the intensity of helminth infection or differences in the infecting helminth species. Helminth burden has been correlated with HIV-1 RNA levels in HIV-1 co-infected individuals and may be an important factor in determining the extent to which helminths affect HIV-1 progression [2]. In addition, helminth species differ in their level of tissue invasiveness, level of resulting host immune activation and other factors which may explain observed differences in the interactions between HIV-1 and various helminths seen in observational studies [8].

All of the included studies were limited by short follow-up duration. The finding of changes in HIV-1 RNA levels despite the short duration of follow-up in these studies is perhaps more biologically plausible than a finding of changes in clinical staging or CD4 counts. Helminth infection may directly suppress the Th1 response leading to a reduction in virus specific CD8+ cytotoxic T lymphocytes (CTL's) [28],[29]. Plasma HIV-1 viral load is directly related to HIV-1 specific CTL responses in humans and a reduction in CTL response is associated with a more rapid progression of HIV-1 disease [30],[31],[32],[33],[34]. It is plausible that the changes in immune control of HIV-1 replication could lead to more immediate changes in HIV-1 RNA levels following helminth infection or eradication. In contrast, CD4 decline appears more related to immune activation status and changes in regulatory T cell expression and function which may resolve more slowly following treatment of helminth infection [8].

The ideal study design to determine anti-helminth treatment effect on HIV-1 progression is a randomized clinical trial. Thus, randomization to immediate versus deferred treatment was used in the single RCT of schistosomiasis eradication. However, the short period of follow-up ethically feasible for treatment deferral limits ability to determine longer term effects of anti-helminthics on HIV-1 progression. Randomization without determination of helminth-infection status may be an alternative study design to determine if anti-helminthics should be empirically provided for individuals in HIV-1 care programs in areas of high helminth prevalence. This approach does not require helminth-screening and may enable determination of helminth treatment effects over longer periods but would require sufficient helminth prevalence to achieve power to detect effect.

The results of this systematic review suggest there are insufficient data to determine whether de-worming patients with HIV-1 has a beneficial effect on HIV-1 viral load, CD4 count or clinical progression. There are significant limitations to all of the studies identified and available data do not support empiric anti-helminthic therapy or routine helminth screening of HIV-1 infected individuals. However, the cumulative evidence suggests that de-worming HIV-1 infected individuals may have beneficial effects on plasma HIV-1 RNA viral load. There is need for large randomized controlled trials with longer follow-up duration in order to assess the impact of de-worming on HIV-1 progression in populations with a high prevalence of both helminth and HIV-1 infection.
